# An Epstein-Barr Virus Anti-Apoptotic Protein Constitutively Expressed in Transformed Cells and Implicated in Burkitt Lymphomagenesis: The Wp/BHRF1 Link

**DOI:** 10.1371/journal.ppat.1000341

**Published:** 2009-03-13

**Authors:** Gemma L. Kelly, Heather M. Long, Julianna Stylianou, Wendy A. Thomas, Alison Leese, Andrew I. Bell, Georg W. Bornkamm, Josef Mautner, Alan B. Rickinson, Martin Rowe

**Affiliations:** 1 Cancer Research UK Institute for Cancer Studies, The University of Birmingham, Edgbaston, Birmingham, United Kingdom; 2 GSF-Institut fur Klinische Molekularbiologie und Tumorgenetik GSF-Forschungszentrum fur Umwelt und Gesundheit, Munich, Germany; 3 Munich University of Technology, Children's Hospital, Munich, Germany; University of Southern California School of Medicine, United States of America

## Abstract

Two factors contribute to Burkitt lymphoma (BL) pathogenesis, a chromosomal translocation leading to c-myc oncogene deregulation and infection with Epstein-Barr virus (EBV). Although the virus has B cell growth–transforming ability, this may not relate to its role in BL since many of the transforming proteins are not expressed in the tumor. Mounting evidence supports an alternative role, whereby EBV counteracts the high apoptotic sensitivity inherent to the c-myc–driven growth program. In that regard, a subset of BLs carry virus mutants in a novel form of latent infection that provides unusually strong resistance to apoptosis. Uniquely, these virus mutants use Wp (a viral promoter normally activated early in B cell transformation) and express a broader-than-usual range of latent antigens. Here, using an inducible system to express the candidate antigens, we show that this marked apoptosis resistance is mediated not by one of the extended range of EBNAs seen in Wp-restricted latency but by Wp-driven expression of the viral bcl2 homologue, BHRF1, a protein usually associated with the virus lytic cycle. Interestingly, this Wp/BHRF1 connection is not confined to Wp-restricted BLs but appears integral to normal B cell transformation by EBV. We find that the BHRF1 gene expression recently reported in newly infected B cells is temporally linked to Wp activation and the presence of W/BHRF1-spliced transcripts. Furthermore, just as Wp activity is never completely eclipsed in *in vitro*–transformed lines, low-level BHRF1 transcripts remain detectable in these cells long-term. Most importantly, recognition by BHRF1-specific T cells confirms that such lines continue to express the protein independently of any lytic cycle entry. This work therefore provides the first evidence that BHRF1, the EBV bcl2 homologue, is constitutively expressed as a latent protein in growth-transformed cells *in vitro* and, in the context of Wp-restricted BL, may contribute to virus-associated lymphomagenesis *in vivo*.

## Author Summary

Cancer almost always develops through the cumulative effects of several independent changes in the target cell. For certain tumors, one step in the chain involves infection of the cell with a particular type of virus. The best example is Burkitt lymphoma (BL), a tumor of B lymphocytes which develops through the combined action of a genetic accident leading to uncontrolled expression of the c-myc oncogene and infection with a common herpesvirus, the Epstein-Barr virus (EBV). Recent evidence suggests that, although latent EBV infection can itself drive B cell growth, the virus plays a different role in the context of BL, namely to counteract the naturally poor survival ability of c-myc–expressing cells while leaving their c-myc–driven growth intact. Here we show that EBV achieves this by unexpectedly switching on a viral protein that was thought never to be seen in latent infection; this viral protein resembles one of the cell's own key survival proteins called bcl2. Furthermore, the work has led us to realise that this virally encoded bcl2-like protein is not only important in the context of BL but, contrary to conventional wisdom, is actually part of EBV's natural strategy for B cell growth transformation.

## Introduction

Burkitt lymphoma (BL) is a human tumor of B cell origin whose pathogenesis involves complementation between a defined cellular genetic change, translocation of the c-myc oncogene into an active immunoglobulin (Ig) locus, and a B cell-transforming virus, Epstein-Barr virus (EBV) [1],[2]. C-myc deregulation appears to be the crucial lymphomagenic event, since all BLs worldwide carry a c-myc/Ig translocation, and in model systems, expression of c-myc from such a construct converts B cells to the proliferating BL phenotype [3]–[6]. There is, nevertheless, strong selection for EBV as a complementing agent. Thus all cases of BL in its high incidence (endemic) form are EBV genome-positive, as are 15–85% of the low/intermediate incidence (sporadic) BLs seen elsewhere in the world [7]. However, the virus' role in BL pathogenesis has remained obscure, not least because EBV gene expression in tumor cells does not mirror that of a typical growth-transforming infection.

**Figure 1:**
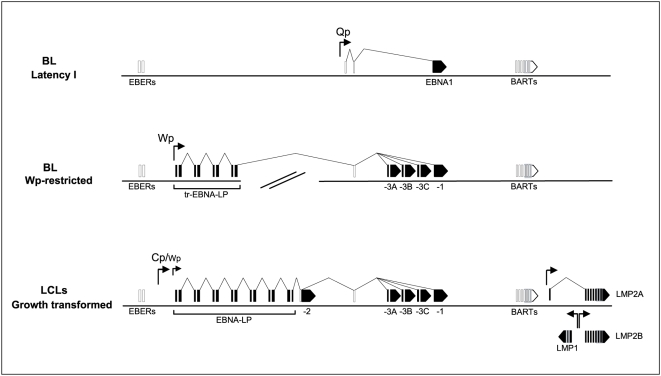
Patterns of EBV latent antigen expression in BL cells and LCLs. EBV is detected in one of two restricted forms of viral latency in BL cells. Most endemic BLs carry a wild-type EBV genome as a Latency I infection involving expression of a single EBV antigen, EBNA1, from the Qp promoter. A subset of 15% endemic BLs carries a mutant EBNA2-deleted EBV genome and expresses a Wp-restricted form of latency. This involves expression of EBNAs 1, 3A, 3B, 3C, and in some cases a truncated(tr) EBNA-LP from a highly active Wp promoter. By contrast, when EBV transforms normal B cells into LCLs *in vitro*, the established lines express a wider range of transforming proteins. This typically involves a highly active Cp promoter (plus low-level Wp activity) leading to expression of EBNAs 1, 2, 3A, 3B, 3C, and −LP, and expression of LMPs 1, 2A, and 2B from their own EBNA2-dependent promoters. Coordinates of the transcription start sites based on the standard B95.8 strain sequence [59] are 11336 for Cp, 14384 for the most 5′ copy of Wp, 62423 for Qp, 166498 for LMP2A, 169740 for LMP2B, and 169128 for LMP1. Note that the non-coding EBER RNAs and BART transcripts are expressed in all the above forms of latency.

To illustrate the point, Figure 1 (bottom line) shows the pattern of latent gene expression established when EBV transforms normal B cells into permanent lymphoblastoid cell lines (LCLs) *in vitro*. This entails expression of the non-coding EBER RNAs, the *Bam*HIA rightward transcripts (BARTs) from which most of the EBV micro(mi)-RNAs are derived [8],[9], six nuclear antigens (EBNA1, 2, 3A, 3B, 3C and –LP) and three latent membrane proteins (LMP1, 2A and 2B). The LMPs are each expressed from their own EBNA2-activated promoters. By contrast, the individual EBNA mRNAs are generated by differential splicing of long primary transcripts, initiated immediately post-infection from the *Bam*HIW promoter, Wp, and later from an adjacent pan-EBNA promoter, Cp [10]. Interestingly, the same cDNA cloning studies that first characterised the EBNA mRNAs also identified rare clones that spliced downstream of EBNA2 into BHRF1 [11]–[13], a gene later recognised as a viral homologue of cellular bcl2 [14]. However the BHRF1 protein could never be detected in tightly latent LCLs and it was subsequently identified as an early lytic cycle protein [15] expressed from its own lytic cycle promoter [12]. More recently, transcription of the BHRF1 gene has been detected in freshly-infected B cells but, because this was transient and accompanied by a number of other lytic gene transcripts, it was thought to reflect opportunistic transcription from the incoming un-methylated virus genome [16].

As also illustrated in Figure 1 (upper lines), EBV-positive BL tumors display quite different forms of latency, with many of the transforming proteins being down-regulated. Most BLs express the EBERs, BARTs and just one protein, EBNA1, from an EBNA1-specific promoter, Qp [17]–[19], a form of infection referred to as Latency I. The resident EBV genome in these tumors is wild-type and transformation-competent [17]. However, we recently identified another subset (around 15%) of BLs where, in addition to EBNA1, the EBNA 3A, 3B, 3C proteins and in some cases also a truncated form of EBNA-LP were expressed, always in the absence of EBNA2 and the LMPs [20]. This reflected the use of a different transcriptional programme, called “Wp-restricted latency”, where the EBNA transcripts all derive from the Wp promoter. Interestingly, another defining feature of “Wp-restricted” tumors was the presence of a mutant EBV genome with a deletion removing the EBNA2 gene and some adjacent upstream and downstream sequences. Note that, although EBNA2-deletion events are sometimes detectable by PCR amplification at sites of lytic virus infection in the oropharynx [21],[22], viruses with such a deletion are defective in transformation and have rarely if ever been detected as latent infections of the normal B cell pool. Therefore, detecting such rare mutant genomes in a significant number of BLs strongly suggests that infection with an EBNA2-deletion mutant (or, more likely, the Wp-restricted latency with which such infection is associated) has markedly increased EBV's potential to act as a cofactor in BL development.

As to what that co-factor role might be, studies of c-myc-driven B cell lymphomagenesis in mouse models have shown that complementary changes often act by counteracting the pro-apoptotic effects of high c-myc expression, thereby giving free rein to myc-driven proliferation [23],[24]. Indeed the first evidence suggesting an anti-apoptotic role for the virus in BL came from work with a Latency I BL line, Akata-BL, where EBV-positive sub-clones proved to be slightly less sensitive to apoptotic stimuli than sub-clones that had lost the EBV genome during *in vitro* passage [25]. This has prompted a large volume of work attempting to identify which Latency I gene products provide a survival advantage to BL cells, with evidence of anti-apoptotic potential being reported for the EBERs [26]–[29], for EBNA1 [30] and most recently for a BART-derived mi-RNA [31] in different experimental contexts. However the levels of apoptosis protection mediated by Latency I infection *in vitro* are relatively slight and the underlying mechanism remains to be fully resolved. It was therefore notable that Wp-restricted BL cell lines were much more resistant to cell death triggers than either EBV-negative or Latency I BL lines [32]. This observation strongly reinforced the idea that EBV's role in BL pathogenesis was to counteract the pro-apoptotic influence of deregulated c-myc expression. Our objectives in the present work, therefore, were (i) to identify the viral gene expressed in Wp-restricted but not Latency I infection, that was responsible for this large increment in apoptosis protection, and (ii) to determine whether the effect was unique to an EBNA2-deleted virus acting in the context of a BL cell or might be highlighting a Wp-associated function that is a natural feature of wild-type virus infections.

## Materials and Methods

### Cell lines

The standard Latency I BL cell lines, Rael-BL, Sav-BL, Kem-BL and Akata-BL, and the Wp-restricted BL cell lines, Sal-BL, Oku-BL and Ava-BL, have been described previously [20], as have the Awia-BL cell line and derived single cell clones (EBV-negative, Latency I and Wp-restricted) and the Awia-LCL [33]. An EBV genome-loss clone of Akata-BL (EBV-loss Akata-BL) was isolated by single cell seeding of the Akata-BL parental cell line. All BL cells were maintained in RPMI 1640 (Invitrogen) containing 10% (vol/vol) selected fetal calf serum and 2 mM glutamine (standard medium), further supplemented with 1 mM pyruvate, 50 µM alpha-thioglycerol and 20 nM bathocupronine disulfonic acid. LCLs, all maintained in standard medium, were generated from the peripheral blood B cells of healthy control donors by infection with wild-type B95.8 strain EBV (WT-LCLs) and with B95.8-derived recombinant viruses lacking an intact BHRF1 gene (BHRF1KO-LCLs) [16] or BZLF1 immediate early lytic gene (BZKO-LCLs) [34]. Note that WT-LCLs typically contain 1–3% of cells in lytic cycle whereas BZKO-LCLs do not contain any lytically-infected cells since the immediate early BZLF1 gene is essential for initiation of the lytic cycle [34]. To provide a reference culture enriched in lytically-infected cells, Akata-BL cells were treated with anti-IgG (Cappell) at a concentration of 0.1% (vol/vol) for 72 hours to induce EBV lytic replication in up to 60% cells [35].

### Plasmids

Inducible gene expression was achieved using pRTS-CD2, a derivative of the pRTS-1 expression plasmid [36]. This plasmid carries a truncated rat CD2 gene, the EBV origin of replication (oriP) and the EBNA1 gene (encoding the viral genome maintenance protein), in addition to a bi-directional doxycycline (dox)-regulated promoter controlling expression of GFP and truncated NGF receptor in one direction and the EBV gene of interest in the other direction. Plasmids were constructed that carried the EBV (B95.8 strain) genes encoding either EBNA3A, EBNA3B, EBNA3C or BHRF1. The BHRF1 construct contained a minimal cDNA with no other flanking EBV sequence; as a non-coding control, we also generated a mutated construct (mut-BHRF1) in which the start codon ATG of the above BHRF1 cDNA had been changed to TAG.

### Generation of stable cell lines carrying regulatable expression plasmids

The pRTS-CD2 derived expression plasmids (10–15 µg DNA) were electroporated, either alone or in combinations, into 10^7^ Sav-BL, Akata-BL and EBV-loss Akata-BL. Cells were allowed to recover in culture overnight before isolating viable cells by density centrifugation followed by separation of rat CD2-expressing transfected cells by magnetic cell sorting using OX34 anti-rat CD2 antibody and MACS anti-mouse IgG_2a/b_ beads (Militenyl Biotech) according to the manufacturer's guidelines. Cultures were expanded and maintained in standard medium. To induce expression of GFP and the gene of interest, dox was titrated into the medium at concentrations from 1 ng/ml to 1 µg/ml for 24 hrs. Typically this procedure yielded cultures in which 30–80% cells stably carried the plasmid; the remaining 20–70% cells lacked the plasmid and served as internal controls. As an additional control in all experiments, Sav-BL, Akata-BL and EBV-loss Akata-BL cells were also transfected with a control plasmid which lacked any EBV gene insert but carried dox-inducible GFP and the truncated NGFR. Cultures were established using the same protocol as above and, following dox-induction, GFP-positive and GFP-negative cells were compared in the same way.

### Cell sorting and detection of EBNA3C-positive cells

In the experiment to demonstrate that GFP expression correlated with expression of the inserted EBV gene of interest, Akata-BL cells stably transfected with the pRTS-CD2 EBNA3C expression plasmid were exposed to 1 µg/ml dox for 24 hrs and then sorted using a FACS Vantage into GFP-positive and GFP-negative populations. These cell populations were smeared onto microscope slides and fixed in ice-cold methanol∶acetone (1∶1 vol/vol ratio) at −20°C for 20 minutes prior to immunofluorescence staining for EBNA3C. The slides were incubated for 30 minutes at 37°C in blocking buffer (1×PBS containing 10% heat inactivated normal goat serum) to prevent non-specific antibody staining, before being stained for 1 hr at 37°C with an antibody specific for EBNA3C (E3CA10 [37]) used at a concentration of 5 µg/ml diluted in blocking buffer. Cells were washed three times in 1×PBS and then stained with a goat anti-mouse Alexa Fluor fluorochrome 594 conjugated secondary antibody (Invitrogen) at a dilution of 1 in 1000 in blocking buffer. Cells were washed three times in 1×PBS, mounted in VECTASHIELD medium containing 4′,6 diamidino-2-phenylindole (DAPI) (Vector Labs) before being visualised on a epifluorescence microscope.

### EBV gene expression

Immunoblotting was carried out as described previously [20] using mAbs to: EBNA1 (1H4), EBNA2 (PE2), EBNA3C (E3CA10), LMP1 (CS1-4), BZLF1 (BZ-1) (all used at dilutions of 1 in 50) [20]; BHRF1 (5B11: Millipore, used at a dilution of 1 in 1000), Calregulin (H-170, Santa-Cruz Biotechnology, used at a dilution of 1 in 1000) and polyclonal antibodies specific for EBNA3A and 3B (Exalpha Biologicals, Maynard, MA; the antibodies were used at a dilution of 1 in 1000 to detect EBNA3A and 1 in 500 to detect EBNA3B) and for PARP1 N-terminal region (H-300, Santa-Cruz Biotechnology, used at a dilution of 1 in 1000). All immunoblots were repeated several times on different protein samples.

To quantify mRNA expression, total RNA extraction and cDNA synthesis was carried out as described previously [38]. Quantitative Taqman (Q)RT-PCR assays specific for Wp-initiated, Cp-initiated, Qp-initiated, EBNA2 and LMP1 latent mRNA transcripts and for BZLF1 (immediate early) and gp350 (late) lytic transcripts are described previously, as are the cell lines used as positive controls for each assay [38],[39]. In addition, expression of the early lytic gene BMLF1 was assayed using a new QRT-PCR assay involving a cDNA primer (5′-GAGGATGAAATCTCTCCAT-3′) and the primers (5′- CCCGAACTAGCAGCATTTCCT-3′) and (5′-GACCGCTTCGAGTTCCAGAA-3′) with a FAM labelled probe (5′-AACGAGGATCCCGCAGAGAGCCA-3′). To quantify BHRF1 expression, we designed two assays using a common cDNA primer (5′-TTCTCTTGCTGCTAGCT-3′), reverse primer (5′-TCCCGTATACACAGGGCTAACAGT-3′) and FAM labelled probe (5′-AATAGGCCATCTTGCTCTACAAGATCTGGCA-3′) all within the BHRF1 coding HF exon, but in combination with one of two different forward primers. Latent BHRF1 transcripts were detected using a forward primer either in the Y2 exon (5′-GAGGATGAAGACTAAGTCACAGGCTTA-3′) or in the W2 exon (5′-TGGTAAGCGGTTCACCTTCAG-3′). Note that both of these upstream primers will detect latent BHRF1 transcripts in WT-LCLs, but only the W2 primer will detect latent BHRF1 transcripts in Wp-restricted lines where the deletion has removed the Y2 exon. A standard LCL with 3% of cells in lytic cycle was used as the positive control for the RT-PCR assays detecting lytic BMLF1, BZLF1 and gp350 transcripts and was assigned an arbitrary value of 1. For quantifying the latent W2-BHRF1 and Y2-BHRF1 spliced transcripts an LCL derived from a lytic cycle-deficient BZKO virus was used as a positive control cell line and assigned an arbitrary value of 1. All QRT-PCR assays were carried out in triplicate and all experiments were conducted on at least three occasions.

### Sequencing of the W2-HF PCR product

cDNA was generated as described above using the BHRF1 specific primer. An aliquot of 50 ng cDNA was amplified in a conventional PCR reaction using Expand High Fidelity DNA polymerase (Roche) and the W2 and BHRF1 PCR primers described above. Briefly the cDNA samples were heated to 95°C for 5 minutes before being subjected to 1 minute incubations at 95°C, 59°C, 72°C for 35 cycles. The W2-BHRF1 PCR products were loaded and run on an 8% polyacrylamide gel in order to get good separation of the 110–265 base pair products (the size of the product depends upon the splicing pattern of the transcript in the different cell lines, see Figure S3). The most intense bands were excised from the gel and the DNA extracted and purified. The DNA PCR product was sequenced using the W2 and HF primers described above on an Applied Biosystems ABI 3700 automated sequencer (carried out by the Functional Genomics Laboratory at the University of Birmingham).

### Apoptosis assays

For the standard panel of Awia-BL clones, 3×10^4^ cells were seeded into wells of a flat-bottomed 96 well plate and treated with either a final concentration of 0.25–1 µg/ml ionomycin (Sigma) or 2.5–10 µg/ml anti-IgM antibody (ICN Flow) at 37°C. Following 48 hrs ionomycin treatment or 72 hrs anti-IgM treatment, cells were harvested, washed in 1×PBS and resuspended in 0.5 ml saline (pre-warmed to 37°C). Syto 16 (Molecular probes Europe, Leiden, The Netherlands) was added to the cells at a final concentration of 25 nM and incubated at room temperature for 1 hr, at which time 2.5 µg/ml propidium iodide (Sigma) was added and the cells analysed immediately on a flow cytometer. A two-dimensional dot plot was generated of Syto 16 fluorescence (y-axis) versus propidium iodide fluorescence (x-axis). Syto 16 will only stain viable cells whereas propidium iodide will preferentially enter necrotic cells [40],[41]. Viable cells (Syto 16 +ve, propidium iodide −ve), apoptotic cells (Syto 16 −ve, propidium iodide −ve) and necrotic cells (Syto 16 -ve, propidium iodide +ve) can therefore be distinguished. Data for 5,000 cells was collected for each cell line.

For the Akata-BL (parental and EBV-loss) and Sav-BL cultures stably carrying dox-regulatable expression plasmids, cells were plated out at a concentration of 2×10^4^ cells per well in a flat-bottomed microtitre plate in media alone or media supplemented with an appropriate concentration of dox (1–1000 ng/ml dox). Cells were then incubated overnight at 37°C, 5% CO_2_ for the expression of GFP and the EBV gene of interest to be induced. The cells were then exposed to the apoptosis inducers, anti-IgM (10–20 µg/ml) or ionomycin (5 µg/ml), and apoptosis assayed in GFP-positive and GFP-negative cells within the same population 48–72 hrs later using propidium iodide (PI 2.5 µg/ml) to identify dead cells. In these experiments cells were not dually stained with Syto 16 because this dye is detected by flow cytometry in the same channel as GFP. Cultures were then analysed by flow cytometry immediately for GFP versus PI staining and results expressed as the percentage death induction within the GFP-positive and GFP-negative fractions. All apoptosis assays were carried out on triplicate cultures on each occasion of testing, and each experiment was carried out on at least three independent occasions.

As an additional measure of apoptosis, 1×10^5^ Akata-BL cells were plated out in multiple wells of a 24 well plate. To some wells, dox was added to a final concentration of 500 ng/ml and the cells incubated overnight at 37°C to allow the expression of GFP and the gene of interest to be induced. Ionomycin was then added to all the wells at a final concentration of 5 µg/ml and the cells incubated at 37°C for 18 hrs. The cells were then harvested, washed twice in 1×PBS and the protein extracted. Western blotting was carried out on 20 µg protein and the membranes probed with an anti-PARP 1 antibody specific for the N-terminal region (H-300, Santa-Cruz Biotechnology, used at a dilution of 1 in1000).

### Infection of primary B cells

To analyse events occurring soon after EBV infection *in vitro*, B cells isolated from adult peripheral blood mononuclear cells (PBMCs) by positive selection using M-450 CD19 Dynabeads (Dynal) were exposed to recombinant EBV (WT, BZKO or BHRF1KO virus) at a MOI of 100 overnight at 37°C, then resuspended in fresh media and plated out at a concentration of 4×10^6^ cells per well of a 24 well plate. At each time point (0, 8, 12, 24, 48, 72, 120 hours post-infection) cells were harvested for RNA (4×10^6^ cells) and protein (8×10^6^ cells). All infections were carried out on at least three independent occasions.

### T cell recognition assays

These experiments involved both freshly-infected B cells studied 4 and 8 days post infection and established LCLs as targets, infections being carried out using recombinant WT virus, the BZKO virus or the BHRF1KO virus. In each case cells were isolated from individuals of known HLA type, positive for DRB1*0401 and DRB1*1501 restricting alleles, or (as a control) from donors mismatched for the alleles. Target cells pre-pulsed for 1 hr with 5 µg/ml relevant epitope peptide served as a positive control. To assay T cell recognition standard numbers (2000 cells per well) of CD4^+^ T cells specific for the HLA-DRB1*0401-restricted BHRF1 122-133 epitope (designated PYY, [42]), the HLA-DRB1*0401-restricted EBNA2 11-30 epitope (designated GQT, [43]) or the HLA-DRB1*1501-restricted gp350 61-81 epitope (designated LDL, [44]) were incubated in 200 µl medium in 96-well V-bottom plates with 10^5^ target cells per well. The supernatant medium, harvested after 18 hrs, was then assayed for IFNγ by ELISA (Perbio) in accordance with the manufacturer's protocol. All T cell assays were conducted in triplicate and all experiments on freshly infected cells and on established LCLs were conducted on at least three independent occasions.

### Statistical analyses

The numerical data derived from the QRT-PCR and apoptosis assays were statistically analysed using the computer program GraphPad Prism 4 (GraphPad Software, CA, USA). For the QRT-PCR assays, the normalised values from all the replicates of the Wp-restricted and Latency I BL cell samples were compared using an unpaired student t-test (two-tailed, 95% confidence interval). For the apoptosis assays performed on the Awia-BL cell lines (Figure S1B), triplicate values for each cell line were used and an unpaired student t-test (two-tailed, 95% confidence interval) employed for the following comparisons; EBV-negative BLs to Latency I BLs and Wp-restricted BLs to EBV-negative and Latency I BLs. For the apoptosis assays performed on the cell lines carrying the pRTS-CD2 plasmids, the values for the percentage death induction in the GFP-positive and GFP-negative cells within each population from triplicate cultures were analysed. Since the individual readings were derived from the GFP-positive and GFP-negative cells within the same culture, here we carried out a paired student t-test (two-tailed, 95% confidence interval) to compare death induction in the GFP-positive (plasmid-positive) cells to the GFP-negative (plasmid-negative) cells.

## Results

The strength of protection from cell death offered to BL cells by a Wp-restricted form of infection is best illustrated in the context of an isogenic system. Awia-BL is an endemic tumor with a characteristic t(8∶14) c-myc/Ig translocation from which we were able to isolate Wp-restricted, Latency I and EBV genome-loss clones in early passage [33]. Figure S1A shows an immunoblot of EBV latent protein expression in these cells, indicating that Wp-restricted clones are distinct from Latency I clones in expressing the EBNA3 proteins in addition to EBNA1. Figure S1B shows representative data from experiments in which these same clones are subjected to graded doses of cell death triggers such as B cell receptor ligation (anti-IgM) or an intracellular calcium ionophore (ionomycin). We have previously shown that the cell death being induced in this system is largely via apoptosis, involving caspase cleavage [33]. Clearly, the Wp-restricted cells are resistant to triggering doses (10 µg/ml anti-IgM, 1 µg/ml ionomycin) that induce widespread death in Latency I and EBV-negative cells. By comparison, the protection being offered by Latency I infection in such assays is much less marked, with differential survival of Latency I compared to EBV-negative clones only being seen as a partial effect at lower anti-IgM and ionomycin doses. Such results strongly suggested that one or more of the viral genes that were exclusive to the Wp-restricted form of infection were responsible for a pronounced increment in cell survival capacity. Referring back to Figure 1, the obvious candidates in that respect were the EBNA3 proteins and/or the predicted product of a truncated EBNA-LP coding sequence (i.e. containing the repeat domains encoded by the W1 and W2 exons, but lacking the unique domains encoded by the Y1 and Y2 exons that are always removed by the deletion). In that regard, while Wp-restricted BL lines and clones are consistently EBNA3-positive, many lack detectable expression of EBNA-LP yet still retain strong apoptosis resistance in the anti-IgM and ionomycin assays [32]. Hence, even though truncated EBNA-LP has been associated with anti-apoptotic effects in some systems through an interaction with protein phosphatase PP2A [45], it could not be responsible for the global apoptosis protection observed in Wp-restricted BLs. At this point, therefore, we focused on EBNA3A, 3B and/or 3C as the potential mediators of protection.

### Apoptosis assays following inducible EBNA 3 protein expression in Latency I BL cells

**Figure 2:**
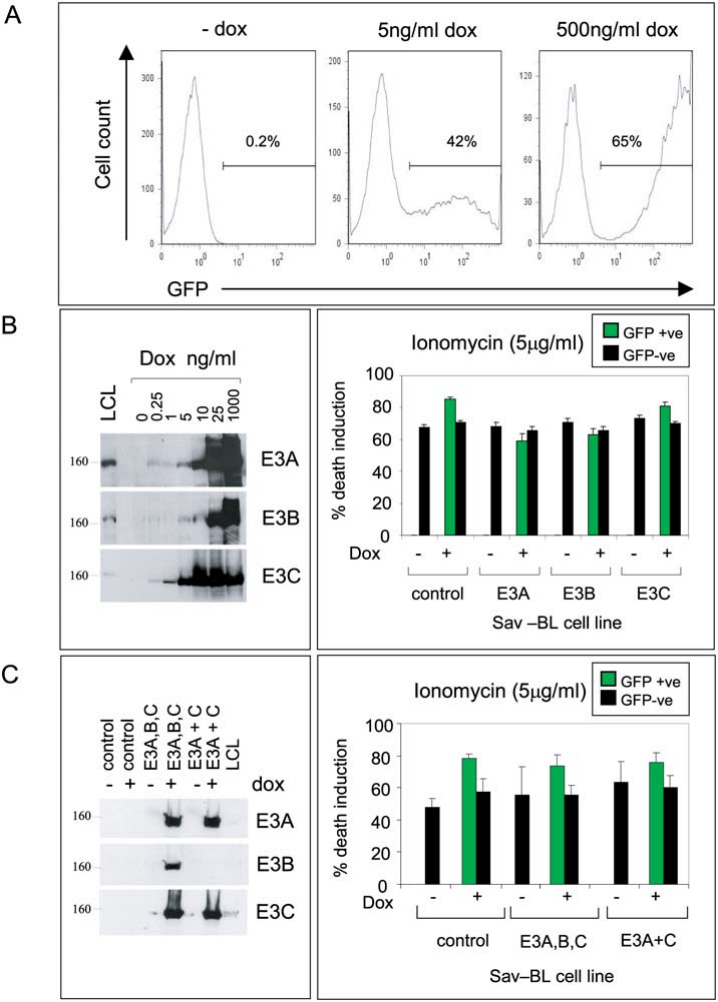
Analysis of the role of the EBNA3 proteins in apoptosis protection. Sav-BL cells were stably transfected with expression plasmids encoding GFP in combination with one or more of the EBNA3 proteins under the control of a bi-directional dox-responsive promoter. (A) FACS profiles showing GFP expression in an EBNA3A-transfected Sav-BL line before and after 24 hr exposure to increasing doses of dox. (B) Left panel shows immunoblotting of either EBNA3A-, EBNA3B-, or EBNA3C-transfected Sav-BL lines following 24 hrs exposure to increasing concentrations of dox; expression levels are compared to those seen naturally in a control LCL. Protein size markers are in kD. Right panel summarises the results of an apoptosis assay on the Sav-BL cells carrying either the EBNA3A, 3B, or 3C plasmids as above or a control plasmid. Cultures of untreated cells or cells induced for 24 hrs with 500 ng/ml dox were exposed to 5 µg/ml ionomycin for 48 hrs, and death induction was detected by PI staining and flow cytometry. In the dox-induced cultures, cell death was separately assayed in the GFP-positive (EBNA3-positive; green bars) and GFP-negative (EBNA3-negative; black bars) cells within the same culture. (C) An equivalent experiment to that described in (B) above, now conducted on Sav-BL cells transfected either with all three EBNA3 expression plasmids, or with EBNA3A and EBNA3C together, or with a control plasmid. Left panel shows immunoblotting for the EBNA3 proteins in these transfected lines following 24 hrs exposure to 500 ng/ml dox. Right panel summarises the results of an apoptosis assay on these same cells, either untreated or induced for 24 hrs with 500 ng/ml dox and then exposed to 5 µg/ml ionomycin for 48 hrs. Results are expressed as in (B) above. Note that the apoptosis assay results in (B) and (C) are shown as the mean percentage death induction+/−SD of triplicate cultures of each type in a representative experiment; cells expressing EBNA3A and/or 3B and/or 3C were not significantly protected from apoptosis compared to control cells. In each case, similar results were obtained in three further experiments of the same type.

In these experiments, we sought to avoid the problems of inter-clonal variability that can beset gene transfection and drug selection experiments in the BL system. Instead we used a new EBV ori-p-based plasmid, illustrated in Figure S2A, which is designed for stable maintenance as an episome in BL cells [36]. Vectored expression of a surface marker, rat CD2, early after transfection allows transfected cells to be enriched, generating a passageable culture in which typically 30–80% of cells carry the plasmid. Thereafter expression of the gene of choice can be induced in a dose-dependent manner by addition of dox. At the same time, dox-dependent co-induction of GFP allows one to distinguish the plasmid-positive cells by FACS staining (Figure 2A). Note, we have confirmed that GFP and the inserted EBV gene of interest are co-expressed in the same cells following induction of the bi-directional promoter with dox (Figure S2B). Initially, two Latency I BL lines (Sav-BL and Akata-BL) were transfected with vectors expressing either EBNA3A, 3B or 3C. Figure 2B (left) confirms that, in each case, expression of the gene of interest is tightly dox-dependent and can be induced either to physiologic (LCL-like) levels or much higher depending on the dox concentration. Following induction, the culture is subjected to apoptotic triggers and subsequently stained with propidium iodide (PI), thereby allowing comparison of the percentage of dead/dying cells in the GFP-positive (EBNA3-expressing) versus GFP-negative (control) fraction. Figure 2B (right) shows data from such an experiment on the Sav-BL background. None of the EBNA3 proteins, expressed individually, offered any apoptosis protection. We then carried out experiments in which the same Latency I lines were co-transfected with the EBNA3A and 3C vectors, since EBNAs 3A and 3C can act cooperatively to alter other aspects of the BL phenotype [46], or with all three EBNA3 vectors. The appropriate combinations of EBNA3 proteins were detectably induced in each case but again showed no evidence of apoptosis protection, either in Sav-BL (Figure 2C) or Akata-BL cells (data not shown).

### Expression of BHRF1 as a latent protein in Wp-restricted BL cells

**Figure 3:**
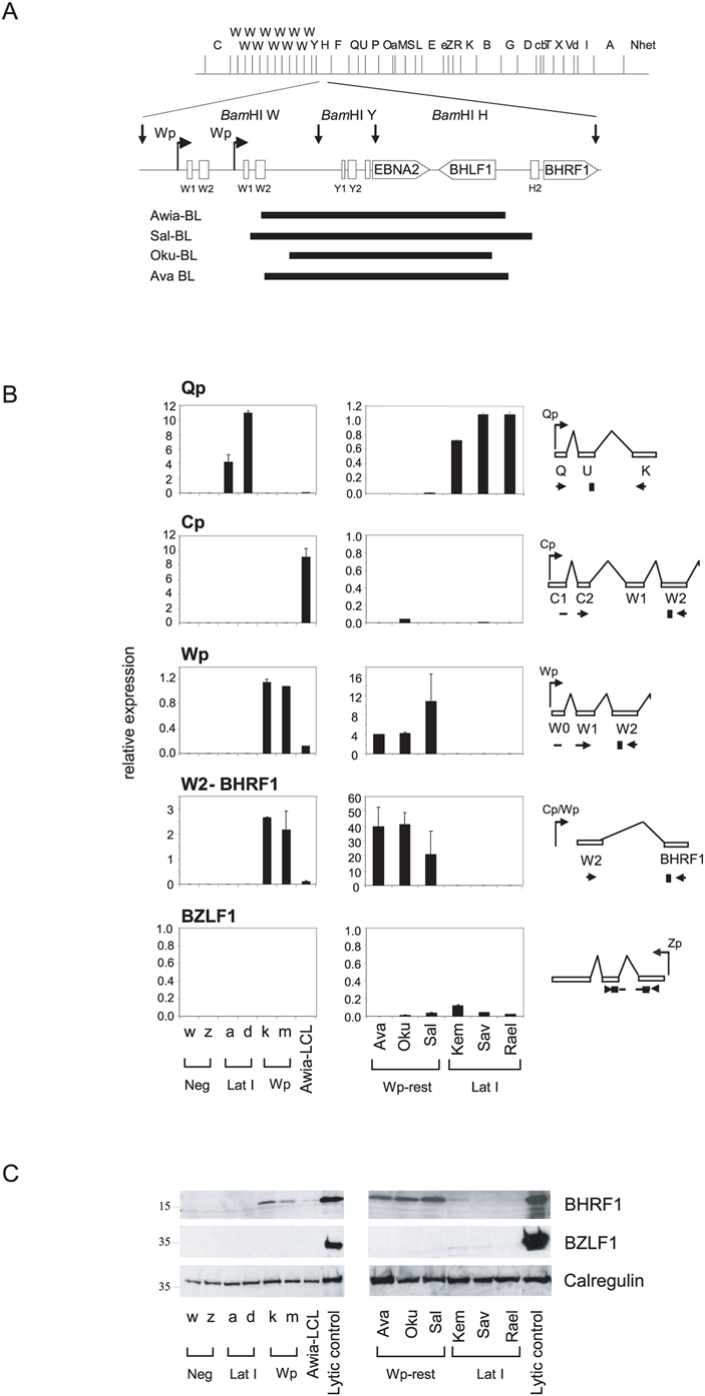
Gene expression analysis of Wp-restricted and Latency I BLs. (A) Linear *Bam*HI restriction map of the EBV genome with a region containing two copies of the *Bam*HI W repeat, and the adjacent *Bam*HI Y and H regions expanded. Wp-restricted BLs carry deletions in that region (see black bars) as described in [20],[33]. These deletions remove part of a *Bam*HI W fragment, all of *Bam*HI Y fragment (including the EBNA2 gene), and part of the *Bam*HI H fragment (including most or all of the BHLF1 gene). (B) Results from QRT-PCR assays used to detect Qp, Wp, and Cp promoter activity, W2-BHRF1-spliced transcripts, and BZLF1 lytic transcripts in (left panel) EBV-negative (w,z), Latency I (a,d), and Wp-restricted (k,m) Awia-BL clones, and (right panel) Wp-restricted BLs, Ava, Oku, Sal, and Latency I BLs, Kem, Sav, and Rael. For each assay, a schematic shows the positions of primer and probe sequences relative to the transcript in question. Results of QRT-PCR assays are shown as the mean+/−SD of triplicate readings. In each case, absolute values were normalised with reference to a GAPDH cellular transcript control and expressed relative to the normalised value from an appropriate positive control cell line which had been assigned an arbitrary value of 1. The results shown are from one representative experiment. Transcripts initiating from Qp were significantly upregulated in Latency I BLs compared to Wp-restricted BLs (p<0.0001). Transcripts initiating from Wp and transcripts splicing from the W2 exon into the BHRF1 coding exon were significantly expressed in Wp-restricted BLs compared to Latency I BLs (p<0.005). Similar results were obtained in three further experiments of the same type. (C) Immunoblotting of the same cell lines as in (B) using antibodies specific for BHRF1, BZLF1, and Calregulin. Akata-BL cells induced into lytic cycle by sIgG cross-linking (approximately 60% lytic) were used as a positive lytic control. This result was confirmed in two further experiments on additional samples taken from these lines; size markers are in kD.

In view of these results, we turned to the possibility that viral gene expression in Wp-restricted BL cells was more extensive than first thought and that other anti-apoptotic candidates, perhaps inappropriately expressed as a consequence of the EBNA2 gene deletion, had been missed. In that regard, Figure 3A illustrates the position of the deletions in Wp-restricted BL lines in relation to the EBV genome as a whole. Note that, in the wild-type genome, the whole *Bam*HI W fragment is tandemly reiterated to form a large internal repeat that lies immediately upstream of the *Bam*HI Y fragment containing the EBNA2 gene. While all four Wp-restricted lines analysed (Awia-BL, Sal-BL, Oku-BL, Ava-BL) carry virus genomes with unique deletion boundaries (see Figure 3A, black bars), in each case the deletion extends upstream of the EBNA2 coding sequence into a *Bam*HI W fragment, thereby removing the unique Y1,Y2 exons of EBNA-LP, and downstream into the *Bam*HI H fragment, removing most if not all of the BHLF1 lytic cycle gene. As illustrated, this brings that copy of the Wp promoter which is nearest the 5′ deletion boundary proximal to the previously described lytic cycle gene encoding the viral (v)bcl2 homologue BHRF1 [15].

We therefore designed a QRT-PCR assay for a transcript that splices from the W2 exon (present in all Wp-driven RNAs) into the BHRF1-coding exon. This was then used to look for evidence of such a W2-BHRF1-spliced species in the previously described panel of Awia-BL clones and in other BL lines representative of Wp-restricted and Latency I infections. Figure 3B shows the results of these W2-BHRF1 transcript assays alongside parallel QRT-PCR assays specific for (i) the Qp-driven EBNA1 transcript known to be expressed in Latency I lines, (ii) all Cp-driven transcripts, and (iii) all Wp-driven transcripts. Within the BL cell panel, the Wp-restricted BL lines and clones were, as expected [20], distinguished by high Wp usage in the absence of either Qp or Cp activity; importantly, these same Wp-using cells also expressed correspondingly high levels of the W2-BHRF1-spliced transcript. For all four Wp-restricted BL tumors, the products of RT-PCR amplification with the W2 and BHRF1 primers were then sequenced to determine their splice structure. As fully described in Figure S3, the structures were slightly different in each tumor depending upon the position of the 5′ deletion boundary relative to the W1 and W2 exons and the position of the 3′ boundary relative to the H2 exon that lies immediately upstream of BHRF1. However, all transcripts spliced from the *Bam*HI W fragment into the BHRF1-coding exon.

We therefore screened the same cell line panel for the presence of BHRF1 protein by immunoblotting with the specific mAb 5B11. As illustrated in Figure 3C, the Wp-restricted BL cells did indeed express the protein, although at levels below that seen in a reference track made from a culture enriched in lytically-infected cells; note that, in lytic cycle, BHRF1 is abundantly expressed from its own lytic cycle promoter situated just upstream in the H2 exon [12]. To counter the possibility that BHRF1 expression in Wp-restricted BL lines simply reflected the presence of a few cells spontaneously entering lytic cycle, we screened these same lines by QRT-PCR assay for transcription of the immediate early lytic gene BZLF1 (Figure 3B) and by immunoblotting for BZLF1 protein (Figure 3C), both sensitive indicators of lytic cycle activity. There was no evidence of such activity, strongly suggesting that BHRF1 is indeed being expressed as a latent protein in Wp-restricted BL cells.

### Inducible BHRF1 expression protects Latency I BL cells from apoptosis

**Figure 4:**
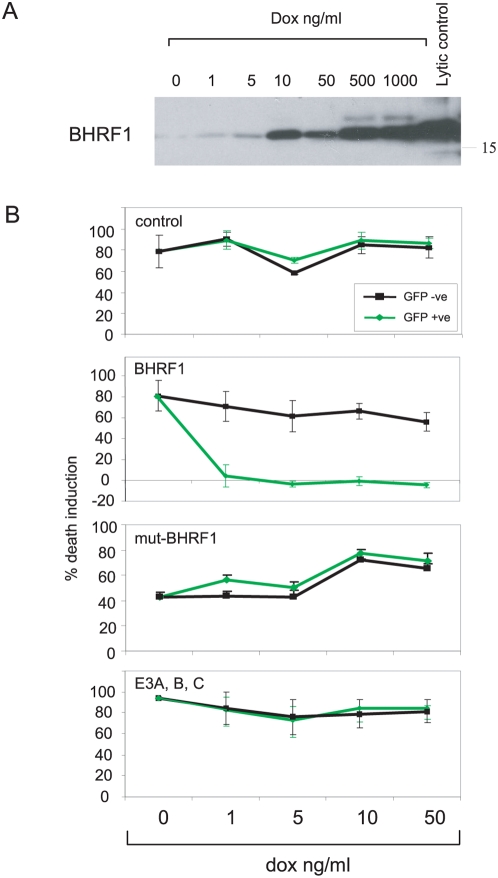
Analysis of the role of BHRF1 in apoptosis protection. (A) Akata-BL cells transfected with the dox-regulated BHRF1 cDNA were either left untreated or induced for 24 hrs with different dox concentrations as shown, then BHRF1 expression assayed by immunoblotting. Akata-BL cells induced into lytic cycle by IgG cross-linking (approx 60% in lytic cycle) were used as a positive control. This result was confirmed in two further induction experiments with this line. Size marker is in kD. (B) Akata-BL cells transfected either with the empty dox-inducible vector (control), or with dox-inducible vectors carrying the BHRF1 cDNA or an ATG-mutated BHRF1 cDNA (mut-BHRF1), or co-transfected with dox-inducible vectors expressing EBNAs 3A, 3B, and 3C (E3A, B, C) were either left untreated or induced for 24 hrs with graded doses of dox before exposure to 5 µg/ml ionomycin for 48 hrs. Cell death was measured by PI staining in the GFP-negative (black lines) and GFP-positive (green lines) cells within the same culture. Results are shown as the mean percentage death induction+/−SD of triplicate cultures of each type in a representative experiment; cells expressing BHRF1 were significantly protected compared to controls at all dox doses (p<0.0001), cells expressing other constructs were not (p>0.05). In each case, similar results were obtained in two further experiments of the same type.

We then asked whether expressing appropriate levels of BHRF1 protein in Latency I BL cells would be sufficient to confer the marked resistance to apoptotic triggers characteristic of Wp-restricted BL cells. In this regard, recent work has identified three EBV miRNAs whose expression is associated with BHRF1 transcription and whose coding sequences lie close to, but outside, the BHRF1 protein-coding sequence [8],[9],[47]. Therefore, to avoid any possible contribution from these or other as-yet-undiscovered miRNAs from this region, the following experiments used an expression construct containing only the BHRF1 cDNA sequence and, as a control, the same construct with a mutation in the initial methionine codon (mut-BHRF1). These constructs were cloned into the dox-regulatable vector and introduced into two Latency I BL lines, Akata-BL and Sav-BL. Figure 4A confirms induction of BHRF1 protein expression in Akata-BL cells carrying the wild-type BHRF1 coding sequence; note that with an inducing dose of 1 ng/ml dox, BHRF1 expression was similar to that seen in the Wp-restricted Awia-BL clones whereas, at 1 µg/ml dox, it approached the much higher levels seen in EBV lytic cycle. These BHRF1 transfectants, plus control transfectants carrying either the mut-BHRF1 sequence or an empty vector and also the previously described EBNA3A, 3B, 3C Akata-BL transfectants, were then exposed to different dox concentrations before assaying for sensitivity to a 5 µg/ml ionomycin challenge. As shown in Figure 4B, the wild-type BHRF1 transfectants were completely protected even at the lowest level of BHRF1 expression whereas the other three types of transfectant remained as sensitive as the co-resident non-transfected population. Equally efficient protection from ionomycin- and anti-IgM-induced apoptosis was also mediated by BHRF1 in the Sav-BL cell line (data not shown). To ensure that this effect of BHRF1 was also apparent in an EBV-negative BL cell background, we generated the same panel of transfectants in EBV-loss Akata-BL cells and obtained an exactly similar pattern of results, as shown in Figure S4A. Throughout these experiments we also confirmed that the anti-IgM- and ionmycin-induced cell death was occurring predominantly by apoptosis, with typical PARP cleavage detectable in dying cells and protection from that cleavage in cells induced to express BHRF1 (Figure S4B).

### Expression of BHRF1 as a latent protein in B cell growth–transforming infections: Physiological relevance of the Wp/BHRF1 connection

Having observed this connection between Wp activity, BHRF1 expression and apoptosis resistance, we were interested to check its possible relationship to the recent finding that, *in vitro*, BHRF1 is transiently expressed in newly-infected B cells, thereby promoting their survival immediately post-infection [16]. We therefore asked whether the W_2_-BHRF1-spliced transcript seen from an EBNA2-deleted genome in Wp-restricted BL cells might also be expressed in normal B cells following infection with a transforming (i.e. non-EBNA2-deleted) virus.

**Figure 5:**
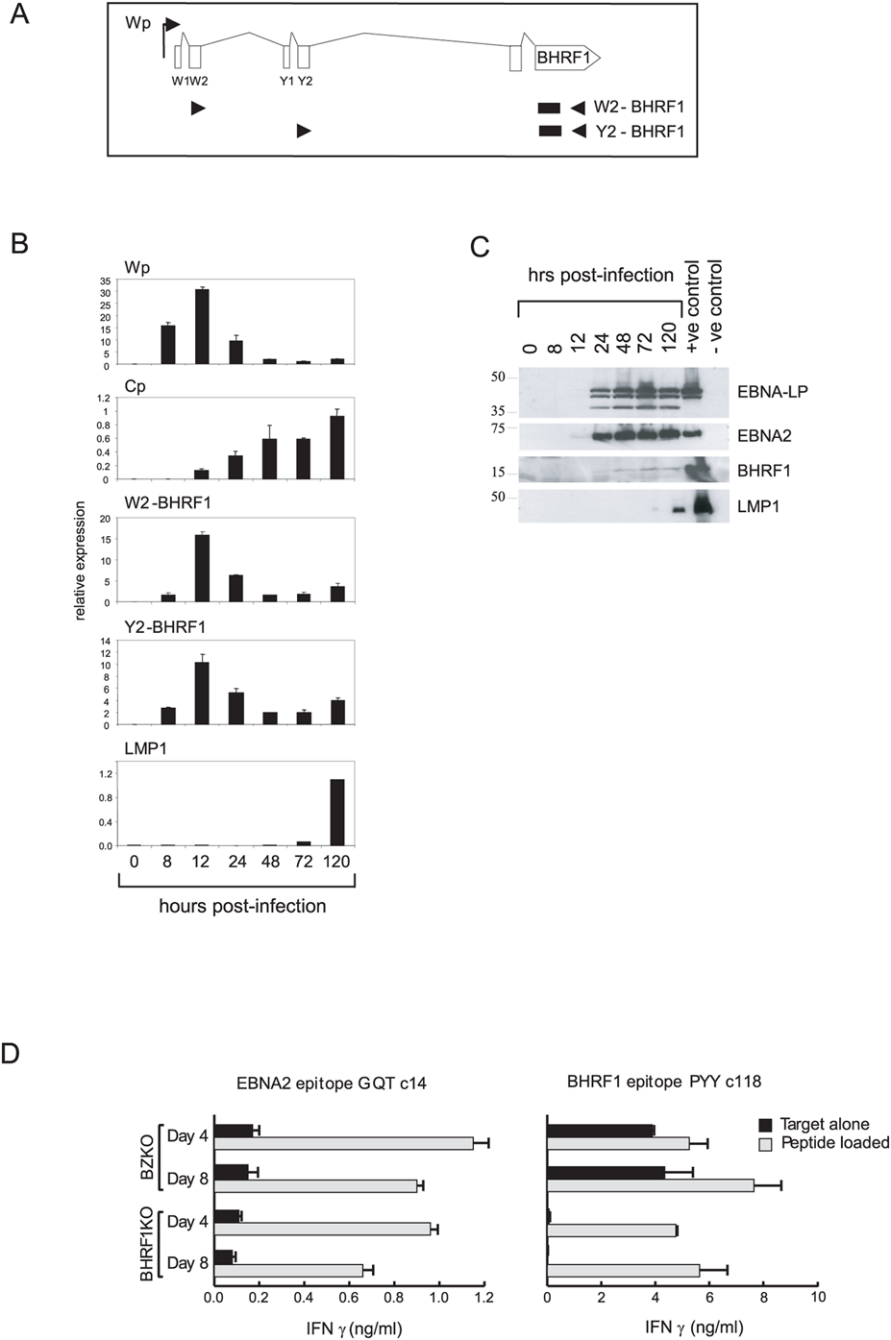
Analysis of early events of *in vitro* B cell transformation. Primary B cells were isolated and infected with the lytic cycle-deficient BZKO rEBV. (A) Map of the latent BHRF1 transcript expressed in LCLs showing the positions of the primers (black arrows) and probe (black box) sequences used to detect W2-BHRF1 and Y2-BHRF1 spliced transcripts by QRT-PCR. (B) Analysis of gene expression using QRT-PCR assays to measure Wp and Cp activity and latent W2-BHRF1, Y2-BHRF1 spliced, and LMP1 transcripts at time points (0 to 120 hrs) following EBV infection. Results, expressed as in Figure 3B, are shown as the mean+/−SD of triplicate readings from a representative experiment. Similar patterns of results were observed in two further infection experiments. (C) Immunoblotting to detect expression of EBNA-LP, EBNA2, BHRF1, and LMP1 at time points following EBV infection. The established LCL X50-7 was used as a positive control for EBNA-LP, EBNA-2, and LMP1 expression. Akata-BL cells induced into lytic replication by surface IgG cross-linking (60% cells in lytic cycle) was used as a positive control for lytic BHRF1 expression. An EBV-negative Awia-BL clone was used as a negative control throughout. Size markers are shown in kD. This result was confirmed in two further infection experiments. (D) CD4+ T cell recognition of HLA-DR4-positive primary B cells 4 and 8 days after infection with either the BZKO virus (lytic cycle-deficient) or with the BHRF1KO virus. Data are shown for (left panel) a DR4-restricted T cell clone specific for an EBNA2 epitope GQT, and (right panel) a DR4-restricted T cell clone specific for a BHRF1 epitope PYY. Results are expressed as IFNγ release into supernatant as measured by ELISA; values (mean+/−SD of triplicate readings) are shown for infected B cell targets (black bars) and for the same target cells pre-pulsed with the relevant epitope peptide (grey bars). A similar pattern of results was obtained in three successive experiments; there was never any recognition of DR4-mismatched targets included as controls in the same assays (data not shown).

In this regard, it is known that Wp is activated immediately following infection, rapidly rises to a peak and then falls as Cp takes its place as the dominant EBNA promoter; LMP1 transcription is EBNA2-dependent and is not seen until after Cp becomes dominant [48]. Since the Wp (and Cp) promoters specifically give rise to RNAs with a W1W2Y1Y2 splice structure [11],[49],[50], it was anticipated that any latent BHRF1 transcript encoded by such a virus would be detectable both by the previously designed W2-BHRF1 transcript assay and by a newly designed QRT-PCR assay using Y2 and BHRF1 primer pairs. The relevant splice structures and primer/probe locations are illustrated in Figure 5A. Normal B cells from healthy donors were therefore exposed to EBV, cultured and then harvested after intervals up to 120 hrs later. Note that, to avoid possible complication from lytic BHRF1 gene expression in these experiments, we used a recombinant EBV strain (BZKO) that had been rendered incapable of lytic cycle entry by deletion of the BZLF1 immediate early gene. Figure 5B shows the QRT-PCR results obtained when virus gene expression in infected B cells was analysed using the W2-BHRF1 and Y2-BHRF1 assays, as well as the standard assays detecting all Wp-initiated transcripts, all Cp-initiated transcripts and LMP1 transcripts. As shown in Figure 5B, W2-BHRF1-spliced and Y2-BHRF1-spliced transcripts were detected as early as 8 hrs post-infection, peaked within 12 hrs and then fell, exactly matching the kinetics of Wp-activity.

Figure 5C shows the results obtained when cells from the same experiment were assayed for protein expression by immunoblotting. EBNA2 and EBNA-LP, the immediate products of Wp transcription, were easily detectable by 24 hr while low levels of BHRF1 were just detectable by 24–48 hrs, some 2–3 days before LMP1. In view of the low levels of BHRF1 detected by immunoblotting, we sought to confirm protein expression by another method. This took advantage of the fact that cells endogenously expressing BHRF1 are efficiently recognised by CD4+ T cells specific for a derived peptide epitope presented by the HLA-DR4 allele [42]. We therefore raised CD4+ T cell clones specific for this BHRF1 epitope from a DR4-positive EBV-immune donor and tested these on autologous B cells after infection with the BZKO virus. As an internal control, we also tested the same target cells for recognition by CD4+ T cells against another DR4-restricted epitope, this time derived from an antigen known to be expressed early post-infection, EBNA2 [43]. The results of these assays are presented in Figure 5D as histograms of interferon-gamma (IFNγ) release; in each case, recognition of infected targets is shown relative to the maximum seen when the same target cells are pre-pulsed with the relevant synthetic epitope peptide. Both sets of antigen-specific T cells showed clear recognition of target B cells at both 4 and 8 days post-infection; indeed the BHRF1 effectors gave the stronger signals. Note that this recognition required *de novo* protein synthesis (rather than antigen acquired from the virus preparation) since DR4-positive B cells assayed immediately after overnight exposure to the virus were not recognised (data not shown). To further check that the BHRF1 effectors were specific, we carried out an equivalent experiment this time using a recombinant EBV (BHRF1KO) in which the BHRF1 gene has been inactivated by insertion of a kanamycin resistance cassette [16]. Cells infected with this virus were indeed not recognised by the BHRF1-specific effectors but were recognised by T cells specific for EBNA2 (Figure 5D). Because all of the above experiments had involved recombinant viral strains, we then repeated the work on cells freshly infected with wild-type virus and obtained a similar pattern of results whether assaying for BHRF1 expression by transcription, by immunoblotting or by T cell detection (Figure S5).

**Figure 6:**
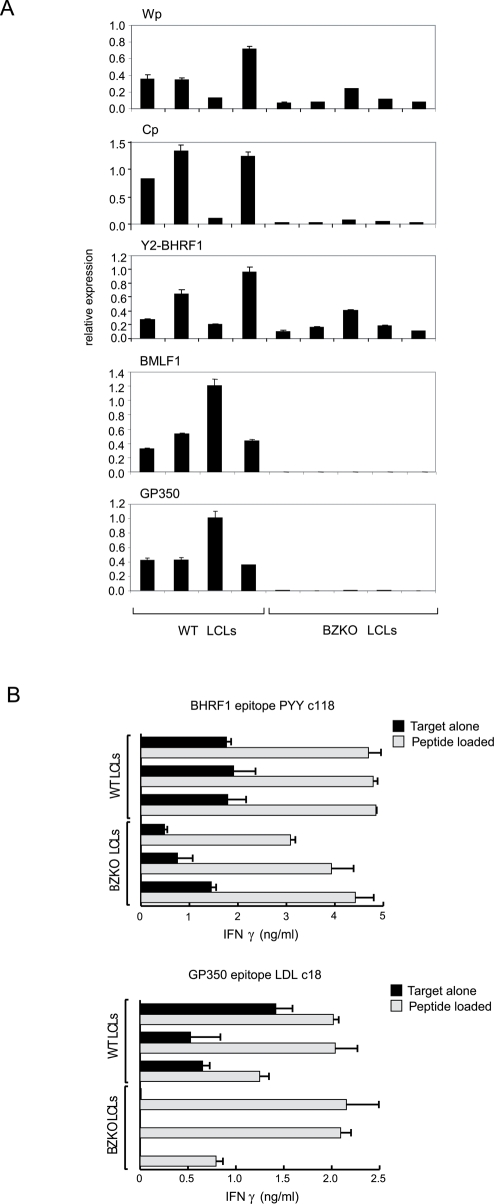
BHRF1 expression in LCLs transformed with either wild-type or BZKO rEBV. (A) Gene expression analysis using QRT-PCR assays to detect Wp-initiated, Cp-initiated, and Y2-BHRF1 latent cycle transcripts, the BMLF1 early lytic cycle transcript and the gp350 late lytic cycle transcript in LCLs transformed either with wild-type EBV or the lytic cycle-deficient BZKO virus. Results, expressed as in Figure 3B, are shown as the mean+/−SD of triplicate readings from a representative experiment. Similar patterns of results were observed in two further experiments. (B) CD4+ T cell recognition of three pairs of LCLs (wild-type and BZKO LCLs) derived from donors expressing both HLA-DR4 and HLA-DR15. Data are shown for (upper panel) a DR4-restricted clone specific for a BHRF1 epitope PYY and (lower panel) a DR15-restricted clone specific for the gp350 epitope LDL. Data are expressed as in Figure 5D as IFNγ release (mean+/−SD of triplicate readings). A similar pattern of results was obtained in three successive experiments; there was never any recognition of DR4-/DR15–mismatched targets included as controls in the same assays (data not shown).

Given that Wp has been shown to remain constitutively active at a low level in all LCLs [51],[52], we went on to ask whether latent BHRF1 expression might persist in the longer term. LCLs were therefore established from a range of donors using both wild-type and BZKO virus strains, then assayed after 2–4 months in culture for latent BHRF1 transcripts, as well as for representative early (BMLF1) and late (gp350) lytic cycle RNAs. Latent BHRF1-spliced transcripts were consistently detected in all LCLs, whether transformed with wild-type or BZKO virus; data from the Y2-BHRF1 QRT-PCR assay are shown in Figure 6A; results from the W2-BHRF1 assay were very similar (data not shown). Sequencing of the W2-BHRF1 RT-PCR products confirmed that they did indeed represent RNAs with the predicted W2-Y1-Y2-BHRF1 splice structure (see Figure S3). By contrast BMLF1 and gp350 transcription was only detected in LCLs carrying wild-type virus, reflecting the presence in these lines of a small percentage of cells spontaneously entering lytic cycle. Interestingly, in all the LCLs, residual Wp activity correlated well with latent BHRF1 transcript levels. However these levels were lower than those seen in freshly-infected cells and, accordingly, BHRF1 protein expression in BZKO LCLs was often at or below the borderline of detectability by immunoblotting (data not shown). However, we reproducibly could detect endogenous expression of the BHRF1 protein in both BZKO and wild-type LCLs by CD4+ T cell assay. Figure 6B shows the results of such assays conducted on pairs of LCLs generated from three different donors; note that these donors were chosen because they were positive both for HLA-DR4, the restricting allele for the BHRF1-specific T cells, and for HLA-DR15, the restricting allele for a CD4+ T cell clone specific for gp350, a late lytic cycle protein. As expected, only wild-type LCLs with some cells in lytic cycle could be recognised by the gp350-specific effectors. However, BHRF1-specific CD4+ T cells consistently recognised both the wild-type and the BZKO LCLs at remarkably high levels, up to 33% of that seen on cells pre-pulsed with cognate peptide.

## Discussion

This work was prompted by the recent finding that, in 15% of endemic BLs, tumor pathogenesis appears to have selected for infection of the target cells with an EBNA2 gene-deleted virus, a transformation-defective mutant that we presume represents only a tiny fraction of the total virus load in the B cell system *in vivo*. Such infection is associated with activation of the Wp latent promoter and a broadening of latent antigen expression beyond that typically seen in Latency I BLs [20]. The fact that this is also accompanied by a marked increase in the tumor cell's resistance to apoptosis [32] immediately helps to explain why such a rare virus mutant had been captured within a disproportionately large number of tumors. This, in turn, greatly strengthens the argument [25] that EBV's role in BL pathogenesis is anti-apoptotic rather than growth-promoting. Here we show that this marked resistance is mediated by Wp-driven expression of the viral bcl2 homologue, BHRF1, a protein hitherto mainly associated with the virus lytic cycle. Furthermore we find that the Wp/BHRF1 connection, though discovered in the context of a mutant virus in BL cells, is also an integral feature of normal B cell infection with wild-type virus. This link with Wp not only explains the burst of BHRF1 expression observed in B cells immediately post-infection [16] but has also led to the finding that BHRF1 remains constitutively expressed as a latent protein in all EBV-transformed LCLs.

We initially focused on the EBNA3 proteins as the most likely mediators of protection from anti-IgM- and ionomycin-induced apoptosis since, at the time, these were the only viral antigens consistently found in Wp-restricted and not in Latency I BL cells. Furthermore in a recent report where EBV-negative BL cells had been stably infected with recombinant viruses (rEBVs) [46], the protection offered by wild-type rEBV infection against cell death induced by nocodazole (which disrupts mitotic spindles), cisplatin (a DNA cross-linking agent) and roscovitine (a cyclin-dependent kinase inhibitor) was lost with virus strains from which either the EBNA3A or EBNA3C gene had been deleted, suggesting that EBNA3A and 3C can act cooperatively to influence the BL phenotype. As a preliminary to the present study, we performed similar infection experiments with EBNA3-knockout viruses on several EBV-negative BL backgrounds but, in our hands, the apoptosis assay results on infected lines proved difficult to interpret because such protocols do not faithfully reproduce Wp-restricted latency. Thus, after the drug selection that is required to establish stable infectants, the selected cells expressed variable levels of viral and cellular anti-apoptotic proteins, namely LMP1 and bcl2, that are never seen in Wp-restricted BL lines (G.L. Kelly, unpublished results). This emphasised the importance of establishing a more controlled experimental system in which to study the effects of specific latent antigens on the apoptosis phenotype. We therefore turned to a novel ori-p-based vector system [36] that allows candidate genes, alone or in combination, to be introduced into Latency I BL lines in silent form and then expression induced to physiologic or supra-physiologic levels by graded doses of dox. This avoids subjecting cells to any drug selection prior to apoptosis assays, and has the added advantage that both vector-positive and control cells exist within the same culture, the former being identified by dox-induction of GFP from the same vector. Using this system, we induced expression of EBNA3A, EBNA3B and/or EBNA3C in two different Latency I cell backgrounds and in an EBV-negative BL backgound, then assayed these cells for sensitivity to triggers of apoptosis (anti-IgM and/or ionomycin) known to distinguish between Latency I and Wp-restricted BL cells. We saw no protection by the EBNA3 proteins, whether expressed individually or together, and whether induced at physiologic levels or much higher.

We therefore began to search for other possible mediators of the anti-apoptotic effect, starting from the observation that all cases of BL presenting with a Wp-restricted pattern of gene expression carried an EBNA2 gene-deleted virus [20],[33]. Close inspection of the different deletions found in individual Wp-restricted tumors showed that each placed a copy of the Wp promoter immediately upstream of the gene encoding BHRF1, a viral bcl2 homologue hitherto thought to be expressed predominantly in lytic cycle [15]. Indeed a W2-BHRF1-spliced RNA could be amplified from all Wp-restricted BL cell lines tested, but never from Latency I lines in which Wp was silent. Moreover a BHRF1 protein could be detected by immunoblotting in all Wp-restricted lines, though at levels much weaker than seen in lytic cycle. Interestingly BHRF1 protein expression and levels of Wp-initiated and W2-BHRF1-spliced transcripts were lower in Awia-BL than in other Wp-restricted lines, perhaps reflecting the single EBV genome copy number in Wp-restricted Awia-BL clones [33]. These results implied that, if ectopic expression of BHRF1 were to explain the apoptosis resistance of these cells, the protein must be active at much lower concentrations than hitherto appreciated. Thus earlier studies had shown that BHRF1 can protect B cells from apoptosis induced by a number of different triggers including growth factor withdrawal [53], TRAIL death receptor signalling [54], γ irradiation and chemotherapeutic drugs [55]. However, such experiments frequently involved vectors giving high level expression. We therefore used the dox-inducible vector system to express BHRF1 in Latency I BL lines at levels ranging from that seen in the Awia-BL clones up to those typical of lytic infection. Remarkably, BHRF1 was fully protective against anti-IgM- and ionomycin-induced apoptosis even at the very lowest level, strongly supporting the view that BHRF1 expressed as a latent protein from the Wp promoter is responsible for the apoptosis resistance of Wp-restricted BLs.

We then went on to ask whether the Wp/BHRF1 connection was unique to the EBNA2-deleted viruses selected for in BL pathogenesis or a hitherto-unrecognised facet of Wp usage in wild-type virus infections. This latter possibility was raised by a recent study showing that BHRF1 was expressed in the first few days following B cell infection with wild-type virus and that this was important for optimal transformation efficiency [16]. There the transient expression of BHRF1 and of a second viral bcl2 family member BALF1, which together appear to protect recently-infected B cells from apoptosis [16], was ascribed to opportunistic transcription from the virus genome following its delivery to the cell nucleus as linear unmethylated DNA. However we found that BHRF1 expression in recently-infected cells was temporally linked to Wp activity and to the detectability of BHRF1 transcripts passing through W2 and Y2 upstream exons. Thus, transcript levels measured by the total Wp, W2-BHRF1 and Y2-BHRF1 QRT-PCR assays all peaked around 12 hrs post-infection and then started to decline as promoter usage switched from Wp to the upstream Cp promoter. These findings suggest that BHRF1 expression is a specific consequence of Wp activation in newly-infected cells and not simply opportunistic transcription from an unmethylated virus genome.

Finally, given recent work showing that Wp is never completely eclipsed by Cp in growth-transforming infections [51],[52], we examined established LCLs for evidence of BHRF1 expression. Both W2-containing and Y2-containing BHRF1 transcripts were consistently detected, whether cells had been transformed with wild-type or lytic cycle-deficient (BZKO) virus. Interestingly some of the earliest analyses of EBV transcription, including work on the tightly latent IB4 LCL, had isolated rare cDNA clones that included W1,W2 and BHRF1 sequences [11]–[13]. However, in the apparent absence of detectable BHRF1 as a latent cycle protein, the significance of the above findings remained obscure. With the advent of more sensitive enhanced chemiluminescent methods for immunoblot detection, we now find that BHRF1 protein is consistently detectable in recently-infected B cells (at times immediately following the peak of Wp activity) and is often just detectable at trace levels in immunoblots of established, tightly-latent, LCLs. The possibility of confirming these observations using T cells, rather than an antibody, as the probe came with the description of CD4+ T cells specific for a defined BHRF1 peptide epitope that recognise target cells endogenously expressing cognate antigen [42]. Here we used the greater sensitivity of these BHRF1-specific CD4+ T cells to show that the protein is indeed constitutively expressed in all established LCLs, even in lines devoid of lytically-infected cells. This puts BHRF1 in a special category of EBV antigens that straddle the lytic/latent divide. Thus it is abundantly expressed from its own promoter in early lytic cycle and also constitutively expressed from a latent cycle promoter in growth-transforming infections.

Setting the present work in its wider context, it is now known that herpesviruses from several different genera have acquired bcl2-homologous genes during their evolution, and express these genes during lytic virus replication, thereby extending survival of the infected cell and maximising virus production [56],[57]. In the case of EBV, and presumably the other gamma-1-herpesviruses of Old World primates [58], the vbcl2 homologue has also been recruited as part of the B cell growth-transforming programme that is unique to these viruses and appears to be important in virus colonisation of the naïve host. This has been achieved by placing BHRF1 under a promoter, Wp, that both initiates transformation and remains constitutively active at some level in transformed cells. While the Wp/BHRF1 connection increases the overall efficiency of B cell transformation [16], it also brings the risk that in other situations inappropriate activation of Wp will lead to unscheduled BHRF1 expression and enhanced survival of the affected cell. We suggest that this is indeed the case in the subset of endemic BLs studied here, where the presence of an EBNA2-deleted virus genome results in high Wp activity and constitutive BHRF1 expression. This draws a direct parallel between the pathogenesis of EBV-positive BL and that seen in mouse models of c-myc-driven lymphomagenesis [23],[24], where the drive towards full malignancy occurs only when a target cell expressing a deregulated c-myc oncogene acquires complementary changes that counteract c-myc-driven apoptosis. The present work suggests that, in a subset of EBV-positive BLs, the complementary factor can be BHRF1. In so doing, it provides the first evidence implicating a herpesvirus bcl2 protein in viral oncogenesis.

## Supporting Information

Figure S1 Latent antigen expression and sensitivity to cell death triggers among Awia-BL clones. (A) Immunoblotting to detect expression of EBV latent antigens EBNA1, 2, 3A, and LMP1 in Awia-BL clones which were either EBV-negative (clones w,z), Latency I (clones a,d), or Wp-restricted (clones k,m); an LCL of normal B cell origin (Awia-LCL) transformed with wild-type Awia-BL virus strain (rescued from Latency I Awia-BL cells) was used as a positive control. Size markers are in kD. Representative results from one of three successive cell samples. (B) Results of assays to detect the percentage death induction in the above clones following exposure to increasing concentrations of anti-IgM for 72 hours (top panel) or ionomycin for 48 hours (bottom panel), as described in Materials and Methods. Results are expressed as mean percentage death induction+/−SD of triplicate cultures of each type. Significant differences (p<0.0001) were observed between Latency I lines and EBV-negative lines at lower but not at higher concentrations of the inducers, and between Wp-restricted lines and both Latency I and EBV-negative lines at the higher concentrations. Results are representative of those seen in two further experiments.
(0.29 MB PDF)

Figure S2 Schematic of the pRTS-CD2 expression plasmid and validation of the system. (A) The expression plasmid pRTS-CD2 carries the EBV origin of replication (oriP) and constitutively expresses the EBV genome maintenance protein (EBNA1) and a truncated rat CD2 protein. In addition, it carries a bidirectional doxycyclin (dox) regulatable promoter (BI-Tet) which on addition of dox to the media drives expression of neuronal growth factor receptor (NGF-R) and green fluorescent protein (eGFP) as markers of plasmid-positive cells, and the EBV gene insert of interest. (B) Immunofluorescence staining for EBNA3C (left panels) and DAPI (middle panels); a merge of the two stains is shown in the right panels. Akata-BL cells stably transfected with the pRTS-CD2 EBNA3C expression vector were exposed to dox for 24 hrs to induce expression of both GFP and EBNA3C from the bi-directional dox-responsive promoter, cell sorted into GFP-positive and GFP-negative populations, and then cell smears of these populations were stained for EBNA3C. All the GFP-positive sorted cells stained positive for EBNA3C (top panels), and all the GFP-negative sorted cells stained negative for EBNA3C (bottom panels), thereby validating the system.
(2.73 MB PDF)

Figure S3 W2-BHRF1 transcript structures in Wp-restricted BL lines. A linear map of part of the EBV genome is shown encompassing two copies of the *Bam*HI W repeat (each with W1 and W2 exons), the adjacent *Bam*HI Y fragment (with Y1 and Y2 exons and the EBNA2-coding exon), and the *Bam*HI H fragment (with the BHLF1-coding exon, the H2 exon, and the BHRF1-coding exon). The exon coordinates, based on the B95.8 strain sequence [59], are 45274-45339 for the most 5′ copy of W1, 45421-45552 for the most 5′ copy of W2, 47761-47793 for Y1, 47878-47999 for Y2, 48386-50021 for EBNA2, 52557-50572 for BHLF1 (only open reading frame currently defined), 53759-53895 for H2, and 54336-55518 for BHRF1. Shown below are the structures of the transcripts amplified by W2-BHRF1 RT-PCR from four Wp-restricted BL lines, relative to the deleted fragment in these same lines; the coordinates of the previously determined deletion [20],[33] are shown. In Awia-BL, the 5′ end of the deletion lies within a W2 exon and the amplified product has a W2-W1-BHRF1 exon structure with some transcripts splicing into the previously recognised position at the start of the BHRF1 exon (B95.8 coordinate 54336) and others splicing further into the exon at a point just 16 nucleotides upstream of the start of the BHRF1 coding sequence (B95.8 coordinate 54360). In Sal-BL, the 5′ end of the deletion lies within a W1 exon and the 3′ end lies within H2; the amplified product has a W2-[truncated W1]-[truncated H2]-BHRF1 exon structure, with the same two entry points into the BHRF1 exon as described above. In Oku-BL, the 5′ end of the deletion is downstream of W2, and the amplified product has a W2-BHRF1 exon structure splicing into the recognised start of the BHRF1 exon. In Ava-BL, the 5′ end of the deletion is in W2 and the amplified product has a W2-BHRF1 exon structure, again splicing into the recognised start of the BHRF1 exon. Note that in all LCLs examined, the W2-BHRF1 amplified product had a W2-Y1-Y2-BHRF1 exon structure, again splicing into the recognised start of the BHRF1 exon.
(0.05 MB PDF)

Figure S4 Analysis of the role of BHRF1 in apoptosis protection. (A) Repeat of the experiment described in Figure 4, now conducted on transfectants of an EBV-loss Akata-BL clone. Transfectants carrying an empty control vector, EBNA3A, 3B and 3C vectors, a BHRF1 vector or a mut-BHRF1 vector were either left untreated or exposed to 500 ng/ml dox for 24 hr prior to challenge with 5 µ/ml ionomycin. Levels of cell death (mean percentage death induction+/−SD of triplicate cultures of each type) are shown for untreated cultures and for the GFP-positive (green bars) and GFP-negative (black bars) cells within induced cultures; cells expressing BHRF1 were significantly protected compared to controls (p = 0.0014), cells expressing other constructs were not (p>0.05). Results are representative of those seen in two further experiments. (B) Analysis of control and BHRF1 transfectants of Akata-BL cells either left untreated or exposed to 500 ng/ml dox for 24 hrs prior to challenge with 5 µ/ml ionomycin. The cells were then harvested 18 hrs later, a protein preparation separated by gel electrophoresis and blotted with an antibody to the N-terminal fragment of PARP1. Cleavage of full-length PARP1 to an N-terminal fragment is indicative of apoptosis. Results shown are representative of two independent experiments; size markers are in kD.
(0.44 MB PDF)

Figure S5 Analysis of early events of *in vitro* B cell transformation. Repeat of the experiment described in Figure 5, now using a wild-type EBV preparation. (A) Analysis of EBV gene expression using QRT-PCR assays of Wp and Cp activity and of W2-BHRF1 transcript levels in primary B cells at time points (0–120 hr) following EBV infection. Results, expressed as in Figure 5B, are shown as the mean+/−SD of triplicate readings from a representative experiment. Similar patterns of results were observed in two further infection experiments. (B) Immunoblotting to detect expression of EBNA-LP, EBNA2, and BHRF1 at the same time points. Control tracks are as in Figure 5C. Size markers are in kD. This result was confirmed in two further infection experiments. (C) CD4+ T cell recognition of HLA-DR4-positive primary B cells 4 and 8 days after infection with wild-type virus. Data are shown for (left panel) a DR4-restricted T cell clone specific for an EBNA2 epitope GQT, and (right panel) a DR4-restricted T cell clone specific for a BHRF1 epitope PYY. Recognition of target cells, with and without pre-pulsing with the relevant epitope peptide, are expressed as in Figure 5D. A similar pattern of results was obtained in three successive experiments; there was never any recognition of DR4-mismatched targets included as controls in the same assays (data not shown).
(0.30 MB PDF)
