# Supplementary figures and images for: An Epstein-Barr Virus Anti-Apoptotic Protein Constitutively Expressed in Transformed Cells and Implicated in Burkitt Lymphomagenesis: The Wp/BHRF1 Link
Source: PLoS Pathog. 2009 Mar 13;5(3):e1000341. doi: 10.1371/journal.ppat.1000341 (PMC2652661; doi:10.1371/journal.ppat.1000341)

A

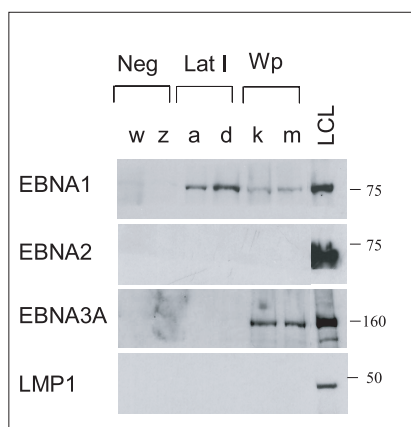

B

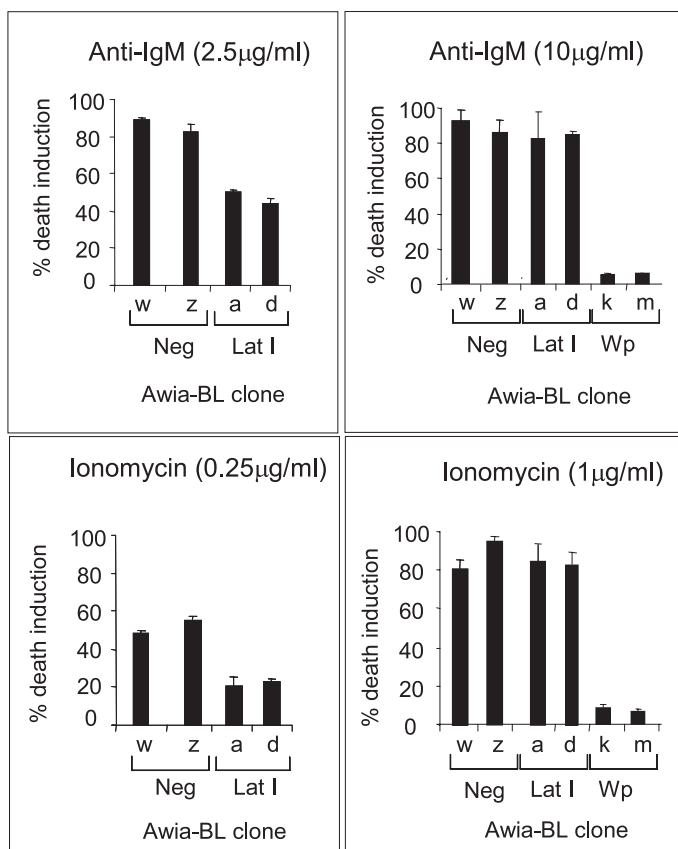

Supplement: Figure S1 — Latent antigen expression and sensitivity to cell death triggers among Awia-BL clones. (A) Immunoblotting to detect expression of EBV latent antigens EBNA1, 2, 3A, and LMP1 in Awia-BL clones which were either EBV-negative (clones w,z), Latency I (clones a,d), or Wp-restricted (clones k,m); an LCL of normal B cell origin (Awia-LCL) transformed with wild-type Awia-BL virus strain (rescued from Latency I Awia-BL cells) was used as a positive control. Size markers are in kD. Representative results from one of three successive cell samples. (B) Results of assays to detect the percentage death induction in the above clones following exposure to increasing concentrations of anti-IgM for 72 hours (top panel) or ionomycin for 48 hours (bottom panel), as described in Materials and Methods. Results are expressed as mean percentage death induction+/−SD of triplicate cultures of each type. Significant differences (p<0.0001) were observed between Latency I lines and EBV-negative lines at lower but not at higher concentrations of the inducers, and between Wp-restricted lines and both Latency I and EBV-negative lines at the higher concentrations. Results are representative of those seen in two further experiments. (0.29 MB PDF) [file ppat.1000341.s001.pdf]

A

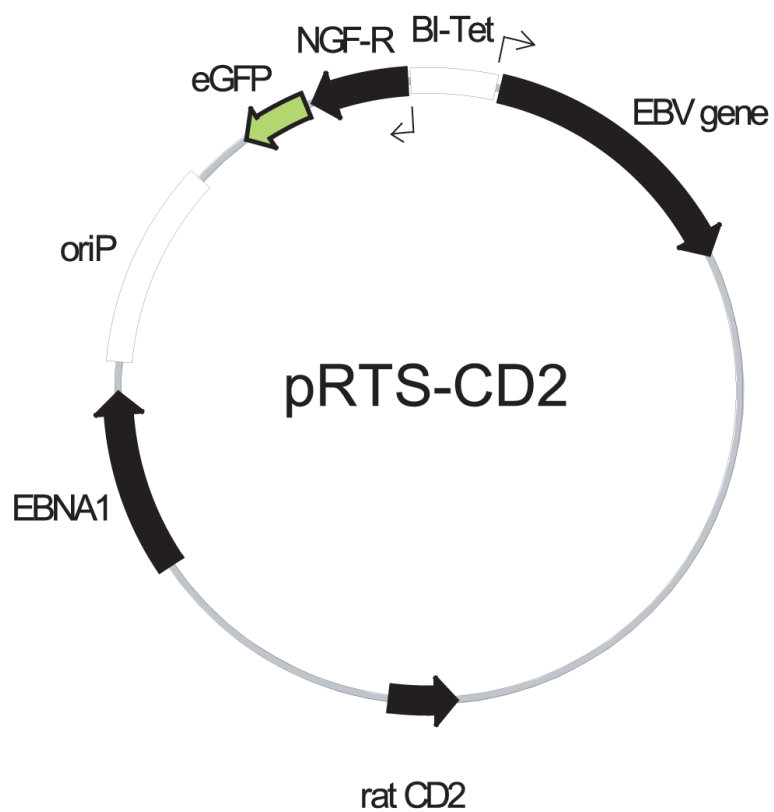

B

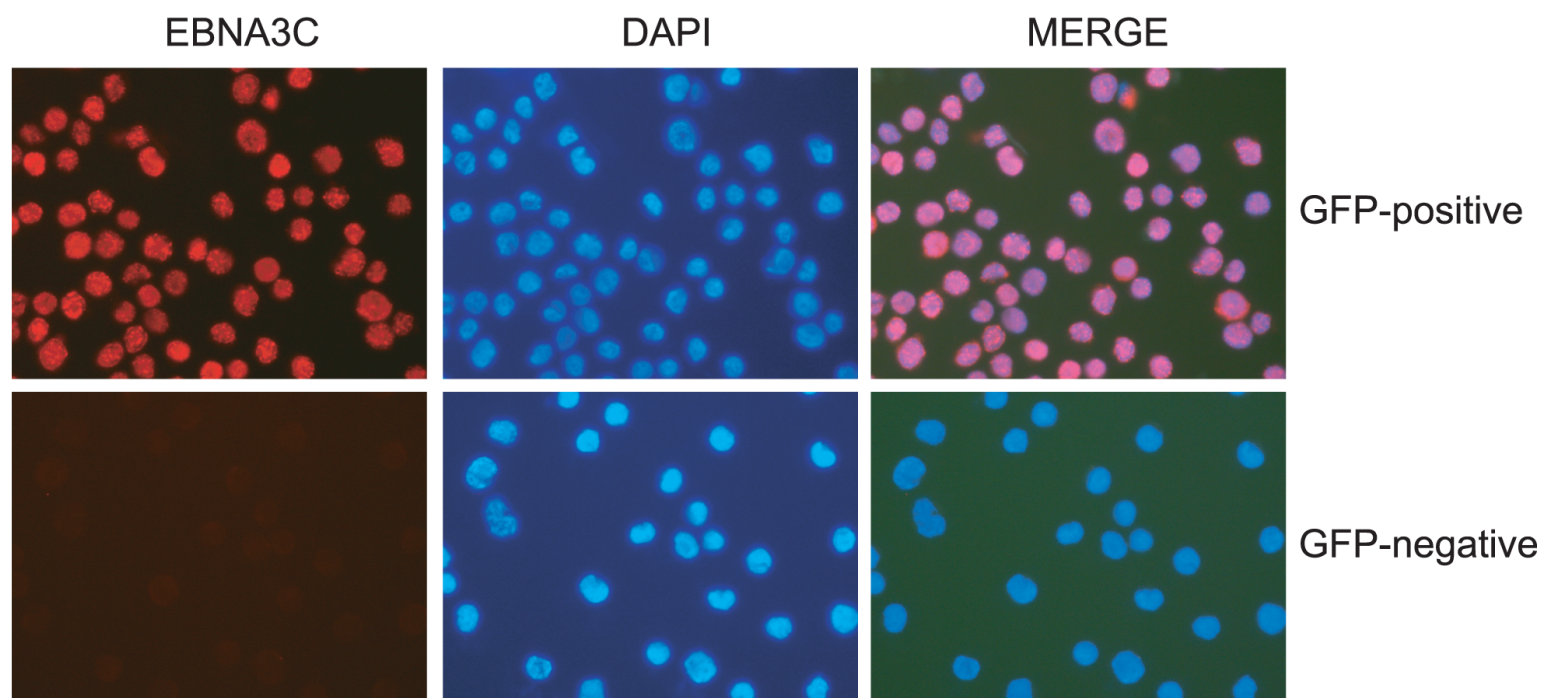

Supplement: Figure S2 — Schematic of the pRTS-CD2 expression plasmid and validation of the system. (A) The expression plasmid pRTS-CD2 carries the EBV origin of replication (oriP) and constitutively expresses the EBV genome maintenance protein (EBNA1) and a truncated rat CD2 protein. In addition, it carries a bidirectional doxycyclin (dox) regulatable promoter (BI-Tet) which on addition of dox to the media drives expression of neuronal growth factor receptor (NGF-R) and green fluorescent protein (eGFP) as markers of plasmid-positive cells, and the EBV gene insert of interest. (B) Immunofluorescence staining for EBNA3C (left panels) and DAPI (middle panels); a merge of the two stains is shown in the right panels. Akata-BL cells stably transfected with the pRTS-CD2 EBNA3C expression vector were exposed to dox for 24 hrs to induce expression of both GFP and EBNA3C from the bi-directional dox-responsive promoter, cell sorted into GFP-positive and GFP-negative populations, and then cell smears of these populations were stained for EBNA3C. All the GFP-positive sorted cells stained positive for EBNA3C (top panels), and all the GFP-negative sorted cells stained negative for EBNA3C (bottom panels), thereby validating the system. (2.73 MB PDF) [file ppat.1000341.s002.pdf]

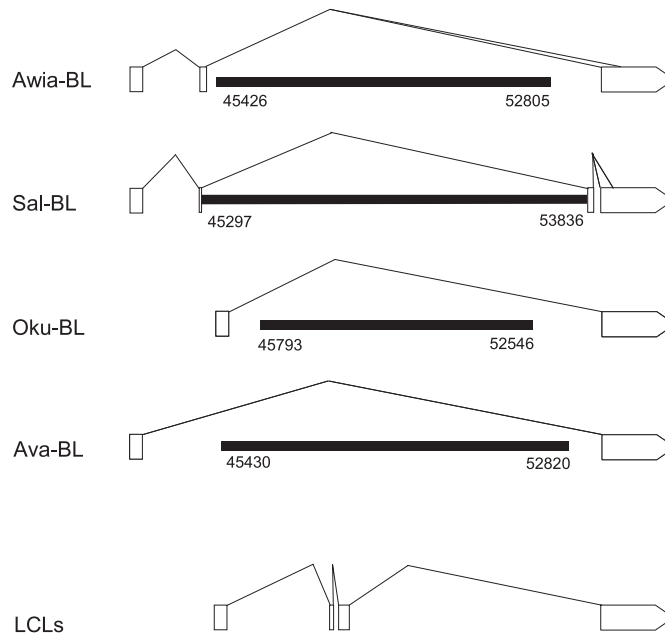

Supplement: Figure S3 — W2-BHRF1 transcript structures in Wp-restricted BL lines. A linear map of part of the EBV genome is shown encompassing two copies of the BamHI W repeat (each with W1 and W2 exons), the adjacent BamHI Y fragment (with Y1 and Y2 exons and the EBNA2-coding exon), and the BamHI H fragment (with the BHLF1-coding exon, the H2 exon, and the BHRF1-coding exon). The exon coordinates, based on the B95.8 strain sequence [59], are 45274-45339 for the most 5′ copy of W1, 45421-45552 for the most 5′ copy of W2, 47761-47793 for Y1, 47878-47999 for Y2, 48386-50021 for EBNA2, 52557-50572 for BHLF1 (only open reading frame currently defined), 53759-53895 for H2, and 54336-55518 for BHRF1. Shown below are the structures of the transcripts amplified by W2-BHRF1 RT-PCR from four Wp-restricted BL lines, relative to the deleted fragment in these same lines; the coordinates of the previously determined deletion [20],[33] are shown. In Awia-BL, the 5′ end of the deletion lies within a W2 exon and the amplified product has a W2-W1-BHRF1 exon structure with some transcripts splicing into the previously recognised position at the start of the BHRF1 exon (B95.8 coordinate 54336) and others splicing further into the exon at a point just 16 nucleotides upstream of the start of the BHRF1 coding sequence (B95.8 coordinate 54360). In Sal-BL, the 5′ end of the deletion lies within a W1 exon and the 3′ end lies within H2; the amplified product has a W2-[truncated W1]-[truncated H2]-BHRF1 exon structure, with the same two entry points into the BHRF1 exon as described above. In Oku-BL, the 5′ end of the deletion is downstream of W2, and the amplified product has a W2-BHRF1 exon structure splicing into the recognised start of the BHRF1 exon. In Ava-BL, the 5′ end of the deletion is in W2 and the amplified product has a W2-BHRF1 exon structure, again splicing into the recognised start of the BHRF1 exon. Note that in all LCLs examined, the W2-BHRF1 amplified product had a W2-Y1-Y2-BHRF1 exon structure, aga [file ppat.1000341.s003.pdf]

A

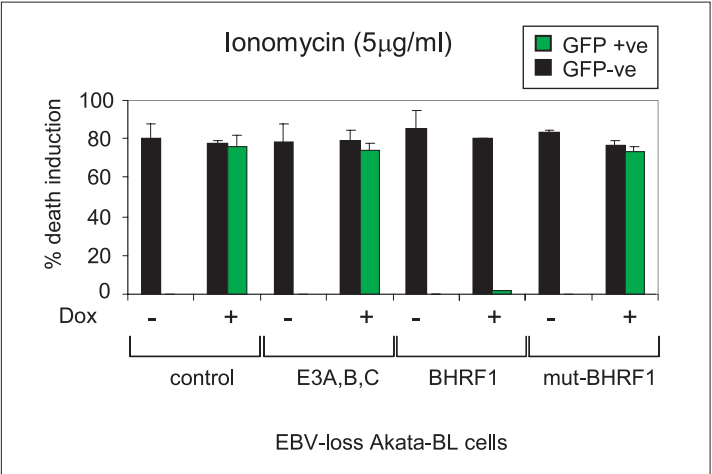

B

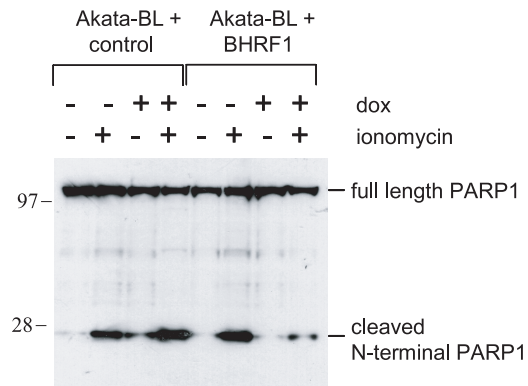

Supplement: Figure S4 — Analysis of the role of BHRF1 in apoptosis protection. (A) Repeat of the experiment described in Figure 4, now conducted on transfectants of an EBV-loss Akata-BL clone. Transfectants carrying an empty control vector, EBNA3A, 3B and 3C vectors, a BHRF1 vector or a mut-BHRF1 vector were either left untreated or exposed to 500 ng/ml dox for 24 hr prior to challenge with 5 µ/ml ionomycin. Levels of cell death (mean percentage death induction+/−SD of triplicate cultures of each type) are shown for untreated cultures and for the GFP-positive (green bars) and GFP-negative (black bars) cells within induced cultures; cells expressing BHRF1 were significantly protected compared to controls (p = 0.0014), cells expressing other constructs were not (p>0.05). Results are representative of those seen in two further experiments. (B) Analysis of control and BHRF1 transfectants of Akata-BL cells either left untreated or exposed to 500 ng/ml dox for 24 hrs prior to challenge with 5 µ/ml ionomycin. The cells were then harvested 18 hrs later, a protein preparation separated by gel electrophoresis and blotted with an antibody to the N-terminal fragment of PARP1. Cleavage of full-length PARP1 to an N-terminal fragment is indicative of apoptosis. Results shown are representative of two independent experiments; size markers are in kD. (0.44 MB PDF) [file ppat.1000341.s004.pdf]

A

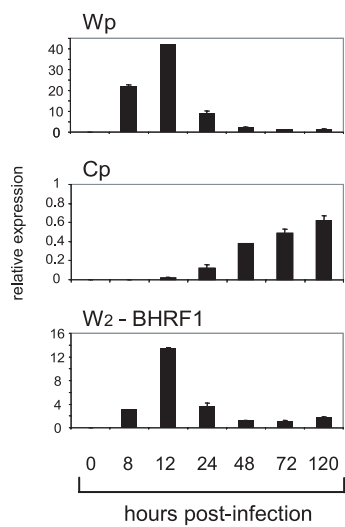

B

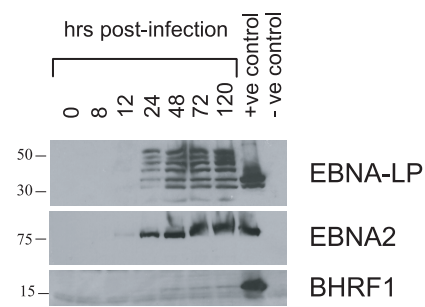

C

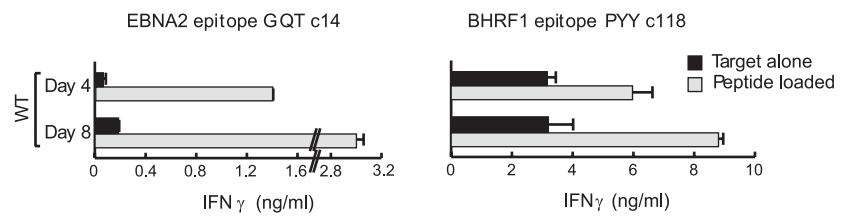

Supplement: Figure S5 — Analysis of early events of in vitro B cell transformation. Repeat of the experiment described in Figure 5, now using a wild-type EBV preparation. (A) Analysis of EBV gene expression using QRT-PCR assays of Wp and Cp activity and of W2-BHRF1 transcript levels in primary B cells at time points (0–120 hr) following EBV infection. Results, expressed as in Figure 5B, are shown as the mean+/−SD of triplicate readings from a representative experiment. Similar patterns of results were observed in two further infection experiments. (B) Immunoblotting to detect expression of EBNA-LP, EBNA2, and BHRF1 at the same time points. Control tracks are as in Figure 5C. Size markers are in kD. This result was confirmed in two further infection experiments. (C) CD4+ T cell recognition of HLA-DR4-positive primary B cells 4 and 8 days after infection with wild-type virus. Data are shown for (left panel) a DR4-restricted T cell clone specific for an EBNA2 epitope GQT, and (right panel) a DR4-restricted T cell clone specific for a BHRF1 epitope PYY. Recognition of target cells, with and without pre-pulsing with the relevant epitope peptide, are expressed as in Figure 5D. A similar pattern of results was obtained in three successive experiments; there was never any recognition of DR4-mismatched targets included as controls in the same assays (data not shown). (0.30 MB PDF) [file ppat.1000341.s005.pdf]
